# “Being the resource is the number one thing”: health professionals supporting trainees’ professional acts of resistance

**DOI:** 10.1186/s12909-025-07169-9

**Published:** 2025-05-07

**Authors:** TingLan Ma, Candace J. Chow, Quang-Tuyen Nguyen, Emily Scarlett, Tasha R. Wyatt

**Affiliations:** 1 Department of Health Professions Education, Uniformed Services, University of the Health Sciences, 4301 Jones Bridge Rd., Bethesda, MD 20814 USA; 2 Department of Internal Medicine, School of Medicine, University of Utah, Salt Lake City, UT USA; 3 Department of Pediatrics in the Spencer Fox Eccles School of Medicine, University of Utah, Salt Lake City, UT USA

**Keywords:** Faculty support, Boundaries, Professional resistance, Advocacy

## Abstract

**Purpose:**

In health professions education (HPE), trainees’ resistance against structural harm and social injustice has gained prominence. However, understanding faculty perspectives on supporting such resistance remains limited. This study delves into how HPE faculty conceptualize and support trainees’ resistance efforts, exploring boundaries, rationales, and strategies.

**Method:**

Using constructivist grounded theory, we interviewed 24 faculty members in HPE, including medicine, nursing, pharmacy, and physician assistant. Data were analyzed using open, focused coding, and constant comparative methods. We also drew from conceptual frameworks including tempered radicals and personal space theory to help understand faculty’s conceptualization of boundaries.

**Results:**

We organized the data into four themes. While most HPE faculty acknowledge the importance of supporting trainees, they hold divergent views regarding when to offer such support and how trainees should engage in acts of resistance. We identify four common boundaries—patient safety, professionalism, professional consequences, and personal safety—that influence faculty considerations. within these boundaries, various supporting strategies were employed, including affirming, building mindset against tokenism, and minimizing DEI performative action.

**Conclusions:**

These findings highlight the dual role of faculty in balancing professional standards while fostering a space for trainees’ work, which offer insights for trainees to realign their resistance efforts with these boundaries.

**Supplementary Information:**

The online version contains supplementary material available at 10.1186/s12909-025-07169-9.

In the past few years, professional resistance has emerged as a significant force in health professions education (HPE) to center social justice, equity, diversity, and inclusion (JEDI) [1–3]. Trainees resist social harm and injustice in their educational and clinical environments in an attempt to bring forth a new vision for health professions education and what the profession can achieve. In large part, their acts of resistance in both overt forms (i.e., protests, walkouts, etc.) and *everyday* forms (i.e. noncompliance, foot dragging, etc.) include speaking out about unfair and toxic working conditions and the policies and structures that result in mistreatment [4, 5]. 

While previous studies indicate trainees’ efforts are supported by faculty members working in the shadows [3], we still do not have a clear picture of when and how faculty support resisting trainees. Specifically, it is unclear whether faculty support trainees in every instance or only in certain circumstances, and whether their support is limitless or if faculty impose boundaries for themselves and others. Understanding where faculty set boundaries as they support trainees clarifies what the HPE community will tolerate when they engage in resistance. To address this gap, this study examines the boundaries that HPE faculty set for themselves and for trainees as they work to create transformational change in health professions education.

## Professional resistance in trainees

Since 2020, there has been a sharp increase in health professions trainees’ efforts to address social harm and injustice in both educational and clinical settings [6]. Among their efforts, they have raised critical questions on how larger societal issues affect care, the disproportionate impact of structural racism on health, healthcare disparities faced by racially minoritized communities [7], and insufficient support against discrimination [5]. They have also taken a stand against police brutality, highlighting racial violence as a matter of public health concern [8, 9]. 

While these types of protests have historical roots [10], their recent surge in scale and visibility is noteworthy [11]. To capture these acts, Ellaway and Wyatt conceptualized *professional resistance*: as expressions—individual or collective—condemning social harms and injustices, aimed at halting them, preventing their recurrence, and/or holding those accountable for them [12]. Encompassing trainees’ acts are direct forms such as unionizing, protests, or strikes, but also more subtle forms such as nonconformity and non-compliance [13]. Many engage in this work because they are experiencing deep-seated moral distress from the mistreatment of patients and learners [10] to which they challenge established power dynamics and question the accepted norms that allowed these issues to manifest [14]. 

Yet, despite resistance being integral to the responsibilities of health professionals, when trainees partake in such actions, they jeopardize their professional status. Potential repercussions include retaliation, negative evaluations, allegations of insubordination and unprofessional behavior, or even being forcibly removed from the system [11]. Consequently, the effectiveness of trainees’ endeavors to reform the system depends on two critical factors: a) the trainees (a) capacity to identify issues within prevailing power structures, (b) identify avenues for effecting change [11], and (c) the degree of support and encouragement they receive from others (i.e., faculty, staff, administration).

### Faculty supporting resistance efforts

Outside of health professions education, faculty and staff who partner with trainees to create institutional change are known as “tempered radicals” [15]. These are individuals who are committed to their institution and endeavor to create change, yet lack significant formal authority to do so [16, 17]. Kezar et al. [15]. noted faculty members often function in this capacity because they are able to create space for trainees to develop as change agents on issues related to inequity. Faculty can support them by giving them information, strategies, and other resources to achieve their goals. The challenge, however, is that faculty must provide this support without jeopardizing their positions or isolating themselves from others in the institution, which requires setting boundaries [18]. Boundaries help faculty stay aligned with professional values while attempting to support trainees, and maintain their professional commitments to the institution in which they work.

Being a tempered radical requires that faculty members consider the integrity of an individual or group and help them set realistic limits. These limits are psychological demarcations that protect individuals and groups and is best described by personal space theory [19]. The boundaries that faculty help trainees create are used to guide their interactions and help trainees ensure their work stays in alignment with crucial professional values [20]. Some of these values might include professionalism, equity in care, patient safety, and selflessness. By helping trainees understand the boundaries in which they must work, faculty fortify safe spaces for trainees to voice their concerns and craft solutions [21]. 

This study aims to examine the boundaries that HPE faculty establish for themselves and the trainees they support in resistance efforts. Our goal was to understand where these boundaries and how HPE faculty work to maintain them. Specifically, we seek to answer the following questions: (1) What boundaries do HPE faculty create for themselves and the trainees whom they support in resistance efforts? (2) What are their reasons behind these boundaries? and (3) What strategies do they employ? By understanding these boundaries, we hope to provide insights that can help trainees and faculty work collaboratively to address long-standing structural harm and social injustice in medicine.

## Methods

To understand the boundaries around faculty members’ perspectives on supporting trainees’ acts of resistance, we used constructivist grounded theory [22] at the level of design, data collection, and analysis. We chose this methodology because we needed an approach that was flexible and responsive enough in our analysis of when and how faculty support trainees who are resisting.

Participants included 24 HPE faculty members in which thirteen held MD degrees and were from schools of medicine; the others were from departments of nursing, pharmacy, and physician assistant (See Table 1). We recruited across multiple institutions using the team’s personal and professional networks. Recruitment began with the intention of maximizing diversity (e.g., race, gender, specialty, and profession) to ensure varied perspectives and potential issues related to resistance. We then turned to snowball methods, attending to divergent experiences throughout data collection (e.g., ensure we include both instances of supporting and non-supporting).

**Table 1:** Demographic characteristics of HPE faculty participants

Characteristic	Number (%)
**Geographic Location**	
US-Northeast	1 (4)
US-Midwest	2 (8)
US-South	9 (38)
US-West	11 (46)
Other (non-US)	1 (4)
**Profession**	
Medicine	16 (67)
Nursing	3 (13)
Physician Assistant	3 (13)
Pharmacy	2 (8)
**Primary Degree**	
MD	13 (54)
DNP	2 (8)
RN	1 (4)
PA	3 (13)
Pharm-D	2 (8)
PHD	3 (13)
**Age**	
30–39	4 (17)
40–49	9 (38)
50–59	7 (29)
60+	1 (4)
unstated	3 (13)
**Leadership Role?**	
Yes	11 (46)
No	13 (54)
**Gender**	
Male	9 (38)
Female	15 (63)
**Race**	
White	15 (63)
White Jewish	2 (8)
Black	4 (17)
Asian	3 (13)
**Hispanic**	
Yes	1 (4)
No	23 (96)

Using a semi-structured protocol (Appendix A), each participant was interviewed between 45 and 60 min over an online platform (i.e., Zoom and Google Meet) and stopped when we reached theoretical sufficiency [23] and robust patterns were clear. This study was deemed exempt by the Uniformed Services University [DBS.2022.472] and was also approved by the University of Utah [IRB #00161829]. Following the exempt protocol, Implied consent was gathered via email communication and oral consent was obtained before the interview began. Participants’ signatures to the formal consent were waived by the institution IRBs to help protect the confidentiality of our participants.

Interview data were transcribed and analyzed using grounded theory methods, an inductive approach that allows for theory generation and construction [24]. We began with open coding and then moved to focused coding using constant comparative methods. All authors coded the same set of 12 transcripts line by line, analyzing the kinds of rationale faculty refer to when they considered offering support to the trainees. The team met bi-weekly for discussion and constructed an initial set of themes delineating faculty’s various consideration. We then noticed that faculty reiterate the importance of keeping a safe space, which is a construct explained in literature by personal space theory [19]. This theory emphasizes how individuals set boundaries to establish safe limits on their participation in an activity. It also highlights how individuals actively regulate the permeability of these boundaries based on specific social cues of the contexts. To further reflect how these considerations are intertwined with faculty’s personal motivation, we also drew form the framework of tempered radicals (16–17) which delineates faculty’s motivation in supporting student activism. Prior work using this framework [15, 25] underscores the various rationales informing faculty members’ decisions to support trainee resistance (e.g., seeing themselves as gatekeepers in the professions and educators in providing resources and advice). Additionally, we anticipated seeing faculty’s considerations for professional values and culture [18, 20], which reflects their sensitivity to potential risks associated with supporting resistance. Based on these conceptual frameworks as sensitizing concepts, we refined the theme structure and deductively analyzed the remaining transcripts. We then organized the themes to describe faculty’s conceptualization of boundaries in trainee resistance.

The research team consists of five HPE scholars who have worked together on research projects in various combinations over the years. The team is racially diverse, works in various HPE departments, and has different professional backgrounds. TM is a social science researcher focusing on medical trainees’ mistreatment and burnout. CJC, a medical education researcher, explores the formation of social and professional identity within the context of privilege and oppression. QTN, an academic primary care physician and clinical educator, serves a diverse patient population, addressing challenges related to marginalized identities. ES, a HPE researcher, specializes in wellbeing of trainees and professional identity formation. TW, a critical white scholar, investigates power and oppression in medical education, particularly what contributes to trainees’ success as resistors. All team members were involved in the data collection and analytical process to assist with issues of credibility and trustworthiness, two issues that must be considered in qualitative studies [26]. 

## Results

Most faculty acknowledged that mistreatment, inequitable rules, and breaches of professional conduct are situations where trainees should resist, yet felt that boundaries around issues of patient safety and professionalism as key boundaries for trainees to consider. Participants explained that these boundaries must be considered for trainees to minimize their risk of retaliation or being seen as unprofessional. Faculty also felt they needed to create boundaries for the trainees as they do this work. This included creating safe and bounded spaces for trainee resistors to voice their concerns and initiate change within the profession. And finally, faculty also voiced the importance of thinking about their own safety when offering support for resistance, thus setting boundaries for themselves. Below, we elaborate on these findings.

### Boundary of patient safety: *“[Resistance is acceptable] if there was a genuine*,* well-founded safety issue”*

Faculty expressed that trainees play a pivotal role as change agents within the profession because of their ability to see where harm and social justice hide in ways that are outside faculty member’s perception. They view their responsibility as trying to keep pace with trainees’ insights around social issues and position their role as helping trainees to challenge the social harm and injustice trainees see, “I don’t think anything’s off the table” (P24), as long as “they care about it” (P11). From their perspective, trainees’ efforts are critical to catalyzing change because trainees tend to be more socially aware than most institutional leadership:

As each new class comes in, there seems to be more and more change agents, and I don’t want them to lose that part of themselves. So, the more that we can practice and show them that they have agency and that they can, even if they aren’t experiencing it themselves, they can actually be the person there providing active resistance, you know, in for someone else. (P13)

Faculty explained their desire to support trainees stems from multiple sources including their own experiences of policies, training rules, and health professional practices that have been “discriminatory,” “arbitrary”, and “capricious” (P2, P3). Thus, these faculty believed trainees should be encouraged to challenge anything that brings harm, but especially “rules that don’t seem to be equitable” (P11) and various forms of “mistreatment” in they encounter. As this faculty expressed, “I would tell them to resist when it’s a personal slight or professional conduct toward you, or if you feel that you need to stand up for a colleague that might have been mistreated” (P9).

One area in which faculty actively endorsed resistance was around patient safety, which is an area in which everyone in the profession is encouraged to speak up [27]. For example, even one of the faculty members who was less supportive of other kinds of targets of resistance agreed on this point,

If there was a genuine, well-founded safety issue that the institution was ignoring and being unresponsive to, I would certainly be supportive of those efforts. I would support that level of resistance to that institutional policy. (P5)

Others voiced similar thoughts and mentioned they would support trainees “If the goal is to provide better care or help us be better clinicians” (P4). This indicates one of the clear boundaries set by faculty members was around patient safety. If safety was a concern, trainees would receive the support they needed, which may be in part why trainees often tie their motivations to this issue [11]. Likewise, one thirds of interviewed faculty clarified they would not support resistance if trainees’ efforts disrupted patient care, which they felt was a patient safety issues. One faculty member shared, “I would be upset if my residents protest[ed] and walked out on their clinical duties. I would find that to be extremely unprofessional because of the needs of our patients” (P1). Another described the boundary this way, “I guess I want to be very supportive, but the [clinical] work also has to get done. For me personally, I think if patient care is compromised, that is something that.is hard for me to support” (P4).

### Boundary of professionalism: *“it’s how you do it”*

Yet, while faculty generally express support in principle if trainees’ concerns are justified, the level of their support depends on how trainees approach the issue, as stated by this faculty “even if it’s an environmental issue, occupational health, they should be involved; it’s how you do it.” (P11).

In evaluating trainees’ resistance efforts, faculty members considered “professional values” (P24) in this work: “the manner in which the resistance is done is more of where the boundaries are and making sure that we uphold professionalism” (P25). Under this boundary, resistance efforts were less supported if the acts were perceived by faculty as “immature and self-centered from a professionalism perspective” (p5), such as when trainees resist because of their lack of willingness to take on assigned responsibilities that involve working extra hours.

Therefore, to uphold professionalism, some faculty members advocated for a systematic approach to solving trainees’ concerns, preferring feedback mechanisms like student surveys rather than sudden strikes or walkouts (P1, P4). They emphasized the significance of open discussions to address issues proactively (P25). Overall, faculty members expressed a preference for trainees to communicate with them before resorting to massive resistance, allowing for de-escalation of tensions and constructive resolution of concerns without impacting patient outcomes. To them, this was professional resistance.

### Professional consequences as boundaries: *“not all efforts will be seen through the same light”*

However, there are identifiable risks for both trainees and faculty members when trainees resist. Faculty put themselves at risk for potentially losing their positions (P27) as well as fear of retaliation from higher administrative entities (P5). Only when the faculty member found themselves in a secure position did they feel they had “a little more backbone and courage, and be a little more of an in-your-face activist” approach (P5). Until then, not all feel comfortable putting themselves out there to support trainees’ efforts. Those who have not waited to ensure their own security described being accused of “manipulating students” and incidents of being “retaliated against” from higher administration (P11). Apart from institutional retaliation, faculty members also grappled with the potential strain on faculty-student relationships when the faculty member cannot support the trainee, after having supported them in other instances. Trainees do not always know the nuances around when support can be provided or not provided, which can cause trainees to turn on them, as this participant described,

It’s incredibly painful when they target [me or my colleagues and] we are perceived as the authority figure standing in the way of change. There’s no way to kind of convince [the] person targeting you that you, too, want this change. (P24)

When discussing boundaries, faculty pointed out the importance of considering the potential harm it could cause to trainees, which led them to temper some of the approaches. Faculty understand that trainees who engage in resistance might face serious consequences. This could include being seen as unprofessional and facing negative repercussions in evaluations and potentially impacting their future opportunities, such as recommendations for fellowships or jobs (P1, P5). For instance, one faculty member highlighted a situation where an outspoken trainee, a woman of color involved in a diversity, equity, and inclusion panel, faced rejection when considered for a faculty position due to perceived personality traits (P4). For these reasons, one Black faculty member mentioned that at times he had to suggest, out of protection, some of his trainees to “put their heads down” (P3). He noted the situation: “as a first-gen college students who come[s] from poverty is like, I gotta get through this. I wanna keep my head down” (P3).

This indicates the existence of structural barriers that do not place all trainees on equal terms to engage in resistance. For some, being able to engage in resistance is a privilege, while for others, they might feel compelled to prioritize their progress through the system, fearing that involvement could potentially causing immediate harm (P3). As such, faculty need to tailor their approaches, ensuring they don’t unintentionally place trainees in a risky position since not all trainees’ resistance efforts will be “seen through the same light” (P9).

Despite this, faculty underscore the essential support they could provide, leveraging their established position within the system, to stand in solidarity with and support trainees in their resistance endeavors, as observed in this case: “He (a Black trainee resistor) had Black faculty for certain that could support him, and he felt that he could go to his program director more confidently” (P9).

### Bounding space for trainees to resist: *“getting people to realize they can question things”*

Despite the importance of aligning trainees’ resistance with professional values, faculty recognized the need to ensure a safe space for trainees to express concerns. They stressed the significance of being allies because “being the resource is the number one thing that you can do” (P10). Faculty employed various strategies, including affirming trainees’ choices, “I try to be really affirming of that and they have to kind of pick and choose some of their battles”(P4), instilling confidence to question authority, “all the times that are coming to my head are either like getting people to question things and realize they can question things”(P24), and bringing issues to light:

“Bringing out, give them permission, and so what I did is talk with the class, leadership’s and tell them, you know you’re doing the right thing here. Don’t be silent because now, you know, [another trainee], who you spoke to, he’s the one that that was being labeled as not fit…but he’s done great things.”(P6).

In addition to affirming and empowering, faculty recognizes the importance of preparing a mindset for trainees against tokenism to prevent burnout. One expressed that he Intentionally teaches trainees to be careful “of how you can maybe undo racism and how that responsibility doesn’t primarily lie on you to be the prime educator” (P9). Other faculty stressed, besides offering immediate support or be instrumental for trainees’ particular requests, the importance to craft the peripheral factors by enhancing training program’s overall climate for dialogue about diversity, inclusion, equity, and justice. Along this line, some faculty took the endeavor to educate other faculty on DEI needs, emphasizing the need for genuine understanding rather than performative actions in supporting trainees. One faculty illustrated:

You know [other faculty], they are more performative. And so more trainings, you know, we’re trying to create more faculty development around EDI [equity, diversity, inclusion]…. One of the hardest pieces is, how do we get faculty and staff trained on board. And going, taking a deeper dive to get a really good understanding, so that they’re not performative. (P3)

Finally, faculty underscored the importance of safeguarding the learning space and de-escalating the tension to ensure trainees feel comfortable returning to their training after engaging in resistance efforts, as this faculty expressed:

I would probably support them, but more so [in a way of] ‘What can we do to get a resolution here or de-escalate?’, so they can get back.. [or] feel comfortable returning to training because most wouldn’t be doing it...if they continue to remain in a toxic and unsupportive environment. (P9)

## Discussion

In this study, HPE faculty shared their perspectives on supporting or not supporting trainees’ resistance efforts against social harm and injustice in health professions education. Faculty decisions involved navigating boundaries, including upholding professionalism [20], to ensure patient safety and protect the professional reputation for themselves and trainees. While establishing boundaries, faculty strived to craft bounded space that were capable of adapting to changes. This concept is termed “flexible territoriality” by Novak et al., [21] and serves as a protective space, which enables trainees to participate in resistance efforts to voice their concerns, and drive meaningful changes in HPE [21, 25, 28]. This dual approach, balancing professional standards and fostering a space for resistance, is a crucial but frequently overlooked aspect in HPE, and our study is the first to explore the multifaceted considerations guiding faculty in establishing these boundaries in support of trainees’ resistance efforts.

Our research resonates with the tempered radical framework and extends it as well [16, 17]. This framework illustrates how faculty members, committed to their roles within the organization, strategically moderate their actions to safeguard their positions and avoid isolation within the institution [15]. Despite occupying lower positions in the institutional hierarchy, some faculty members we interviewed feel a responsibility to challenge prevalent institutional values detrimental to marginalized groups. However, these faculty members tend to refrain from direct forms of resistance, such as engaging in protests [15]. Instead, faculty employed a tempered approach by crafting a boundaries, serving as a resource, fortifying safe spaces, and enabling resistance efforts to amplify rather than directly participate.

Further, this study extends tempered radical theory by highlighting a key distinction within health professions education: beyond the faculty-student dynamic traditionally emphasized in this framework, there is also the role of patients. This three-way interaction requires faculty to consider not only what is beneficial for themselves and their trainees, but also what is best for patients in moments of advocacy and professional resistance. This additional layer of consideration adds a unique dimension to the existing tempered radical framework, demonstrating how faculty navigate the complexities of institutional constraints while balancing their responsibilities to both learners and patients. In this context, setting boundaries around assisting trainees in resistance efforts is not only a strategic move to ensure professionalism and patient safety, but also a means of protecting trainees from retaliation and burnout [15]. 

While the tempered radical framework provides valuable insights into faculty support for trainees and their motivations, it primarily focuses on cases where faculty support student advocates. It does not account for instances where faculty feel unable or unwilling to provide support, nor does it address the structural and professional constraints that shape these decisions. Our findings contribute to filling this gap. Our study extends prior work by highlighting the nuanced struggles faculty face when weighing their support for trainees against potential professional and personal repercussions. On one hand, effecting change in health professions education carries risk. Specifically, many faculty we interviewed are clinical faculty. The nature of their position often entails limited options for tenure, which may contribute to a sense of job insecurity [29]. Our findings revealed that HPE faculty struggle between supporting trainees and putting their professional and personal standing at stake while guiding trainees in resistance efforts. On the other hand, this struggle is closely tied to the role of professionalism in shaping the dominant culture and institutional processes within health professions education. As a foundational framework, professionalism establishes standards that guide behavior, ensure accountability, and maintain institutional integrity. However, its influence can sometimes make it challenging to fully integrate voices advocating for change. By exposing these tensions, our study broadens the tempered radical framework to account for faculty’s constrained agency and the difficult trade-offs they must navigate. A critical question emerges: how can faculty uphold professionalism while remaining adaptable to necessary change? To support faculty in balancing these competing demands—responding to learners’ advocacy for social justice, upholding professionalism, and mitigating professional risk—administrative efforts may consider fostering open discussions, including potentially anonymous ones, that encourage reflection on the role of professionalism in shaping resistance and institutional change.

Further, Eckert et al. [18] revealed an inevitable emotional toll on faculty supporting student activism, which affected their professional stance and identity formation. Balancing boundaries while guiding trainees in their resistance can place stress on faculty who are deeply connected to combating structural harm and social injustice [17]. This can exacerbate systemic oppression and isolate faculty from marginalized backgrounds whose voices may already have been silenced. To navigate this, Lynch et al. [30] suggest involving faculty in reflective practices and mentorship to address these challenges. This aligns with our data, where one of the participants mentioned how reflection clarified their support goals amidst institutional accusations of manipulation.

The implications of this study are twofold. First, concerning support for trainees, our finding around boundaries affecting faculty’s decision offers valuable insights for HPE learners and trainees to reconsider or redefine their approaches to resistance efforts in alignment with these boundaries (e.g., patient safety, professionalism, personal safety) to maximize support for their issues of concern. Second, despite varying degrees of support offered in actual resistance acts, nearly all faculty members interviewed expressed a desire to be aware of and discuss trainees’ frustrations before they escalate. Conversely, previous research [11, 31] indicates that learners may not always feel comfortable disclosing their concerns. This discrepancy suggests a potential misalignment and underscores the importance of reassessing how faculty present themselves to cultivate an environment conducive to open dialogue.

This study has limitations. First, we did not focus on a particular specialty within health professions. Our intention was to glean a comprehensive understanding of faculty perspectives and the diverse manifestations of trainee resistance across various health professions. However, each specialty may have distinct professionalism boundaries that influence faculty support for trainees’ resistance efforts. Given that 67% of our participants came from the medical field, our findings may have limited transferability to allied health professions (e.g., rehabilitation sciences, social work, and healthcare administration). Future research should explore these professions to assess the applicability of our findings and identify any unique boundary-setting practices within these fields. For example, some professions, such as social work, may incorporate foundational training in health advocacy and political action, which potentially affects how faculty navigate and support resistance efforts. Understanding these differences could inform more tailored approaches to fostering faculty-trainee collaboration in addressing structural harm and social injustice in healthcare.

Also, relying on willing participants may have biased perspectives toward those who actively support trainees, potentially overlooking viewpoints of faculty with stricter boundaries. Nonetheless, our data included a range of perspectives, from full support to a more reserved stance. We made efforts to include voices expressing a clear refusal to support trainees’ efforts and the reasoning behind it. Finally, this study did not delineate specific roles faculty play as the go-between the administration and trainees, or outline the specific contexts that may influence these roles. Future research may consider exploring the diverse mechanism or power relationship which enable faculty to support trainees. (e.g., tenure status, years in the institution, and power relationship with higher administration). Last, but not least, this study took place in the U.S. before January 2025, a time preceding a significant shift in institutional and societal support for diversity, equity, and inclusion. In this evolving landscape, resistance remains crucial, but the risks associated with it have also intensified. As the political climate continues to shift, perspectives on best practices and critical considerations for supporting trainees may also evolve, which warrants further elucidation.

## Conclusion

This study reveals divergent views among HPE faculty regarding when and how to support trainees in acts of resistance. Yet, we identified more similarities than differences in the underlying principles (e.g., patient safety) guiding faculty decisions to support trainees. Still, future studies would benefit from delving into the specific strategies employed by faculty within particular clinical or course structures to advocate for social justice in support of trainees.

## Electronic supplementary material

Below is the link to the electronic supplementary material.

Supplementary Material 1

## Data Availability

The de-identified qualitative interview transcript data is available upon request.
