# Supplementary material for: “Being the resource is the number one thing”: health professionals supporting trainees’ professional acts of resistance
Source: BMC Med Educ. 2025 May 7;25:669. doi: 10.1186/s12909-025-07169-9 (PMC12060394; doi:10.1186/s12909-025-07169-9)
Supplement: Supplementary file 1 — Supplementary Material 1 [file 12909_2025_7169_MOESM1_ESM.docx]

**Appendix A. Semi-Structured Interview Guide**

**Before the start of the interview, make sure to communicate the definition of resistance, listed below:**

We are going to provide you with a definition of professional acts of resistance, however you can add in your own ideas. This is a new area of study and it is open to interpretation. For the purposes of HPE, we define resistance as: “*Individual and collective expressions of condemnation of social harms and injustices, with the intent of stopping them, preventing them from recurring, and/or holding those responsible to account*.”

**Interview Questions**

1. Tell me a little bit about yourself. What is your role within the institution and in what ways do you interact with trainees?
2. Were there aspects that you encountered when you were a trainee that you wanted to resist? If so, describe some of these issues.
3. Were you explicitly taught how to resist in a professional setting? If so, what were you told about how to resist?
4. Do you explicitly teach others how to resist? If so, what do you teach them?
5. Have you ever witnessed trainees engaged in professional acts of resistance? If so, describe one of these experiences that you witnessed. If not, why do you think you have not seen resistance?
   1. Tell me about the context. What were these trainees trying to achieve?
   2. Tell me about the trainees’ positionality/subjectivity in the situation (what’s the position of power/identity/intersectionality of the trainee relative to others in the system that may have affected how their efforts were perceived…). How were they perceived by others in the situation? [Probe: Did the hierarchy influence how they were judged?]
   3. Tell me about the interactions they were having with others. In what ways were they trying to undermine power?
   4. What was your reaction to their efforts? Why did you choose to provide/not provide support in this situation?
   5. How do you decide when to support trainees and when not to? Provide an example for both situations.

5 [Alternative Route]. If you have not witnessed trainees engaging in professional acts of resistance, imagine the following examples:

1. Imagine that you witnessed your trainees attempting to change curricular content at your institution. These students have been engaged in on-going complaints, which hasn’t resulted in change and have recently escalated their resistance efforts into protests, and walkouts.
2. Or, imagine you witnessed residents resisting an attending or a charge nurse, whom they think is dangerous and unethical. They have decided that it is worth the professional risk to not follow their orders.
   1. What do you think your reaction would be to their efforts?
   2. Why might you choose to provide/not provide support to these efforts?
   3. How would you decide when to support trainees and when not to?
3. Are there boundaries around what a trainee should be able to resist? When do you think it is appropriate for trainees to resist and when is it not appropriate?
   1. **What professional risks may be involved for them?**
4. Are there some strategies that are better for trainees to use/not use as they engage in resistance? Why is that?
5. Are there some strategies that are better for YOU as a faculty member to help them engage in resistance? Why are some strategies better than others?
   1. Helping with language specificity
   2. Advocating on learners’ behalf when they are not around
   3. Providing opportunities for them to share their concerns
6. Are there some topics or situations that should not be resisted? Why should they not be resisted?
7. In order to equip faculty members with a better understanding of how to support trainees’ resistance efforts, what would you want to know?
8. Demographic Questions
9. Profession
10. Current level of training (MD, PhD, etc.)
11. Institution
12. Age
13. Gender
14. Race
15. Ethnicity
